# Respiratory Syncytial Virus-related Death in Children With Down Syndrome: The RSV GOLD Study

**DOI:** 10.1097/INF.0000000000002666

**Published:** 2020-04-24

**Authors:** Yvette N. Löwensteyn, Emily W. E. M. Phijffer, Juliette V. L. Simons, Nienke M. Scheltema, Natalie I. Mazur, Harish Nair, Louis J. Bont

**Affiliations:** From the * Division of Infectious Diseases, Department of Pediatrics, University Medical Centre Utrecht, Utrecht, The Netherlands; † Respiratory Viral Epidemiology Group, Centre for Global Health Research, Usher Institute of Population Health Sciences and Informatics, University of Edinburgh, Edinburgh, United Kingdom; ‡ Scientific Advisory Board and Chairman, Respiratory Syncytial Virus Network (ReSViNET) Foundation, Zeist, The Netherlands.

**Keywords:** Down syndrome, respiratory syncytial virus, acute respiratory tract infection, mortality

## Abstract

Respiratory syncytial virus (RSV) is a major cause of mortality in children younger than 5 years worldwide. Systematic reviews have shown that Down syndrome (DS) is an independent risk factor for severe RSV infection. We aimed to describe demographic and clinical characteristics of children with DS who died with RSV infection.

**Methods:**

We performed a retrospective case series in which data were shared by individual researchers, research networks and physicians worldwide as part of the RSV Global Online Database study. We included children with DS who died when younger than 5 years of age with laboratory-confirmed RSV infection.

**Results:**

We included 53 children with DS and RSV-related mortality from 20 countries in 5 continents. Five (9.4%) children were from low-income or lower-middle-income countries. Median age at time of death was 6.0 months [interquartile range (IQR): 3.00–12.0]. Thirteen (24.5%) children were born term and had no other risk factors for severe RSV disease. In total, 36 (67.9%) children had congenital heart disease, 8 (15.1%) had chronic lung disease and 1 (1.9%) had congenital immunodeficiency. Duration of hospitalization was significantly longer for children with DS compared with children without DS [median length of stay, 13 days (IQR: 6.8–21.0) vs. 8 days (IQR: 3.0–18.5), *P*=0.005].

**Conclusions:**

One-fourth of children with DS and RSV-confirmed death did not have risk factors for severe RSV disease, indicating that DS is an important risk factor for RSV-related mortality. Age distribution at time of death demonstrates that maternal vaccination would not be sufficient to protect children with DS against RSV-related mortality.

## Abstract 2

Supplemental Digital Content is available in the text.

Respiratory syncytial virus (RSV) is one of the leading pathogens causing lower respiratory tract infections (LRTIs) in infants and young children and is a major cause of mortality in children younger than 5 years of age worldwide.^1–3^ Well-known risk factors for severe RSV disease are prematurity, congenital heart disease (CHD) and chronic lung disease. In addition, our Dutch birth cohort comprising 395 children revealed that Down syndrome (DS) is an independent risk factor of severe RSV-associated LRTIs.^4^ This was subsequently confirmed in several other studies^5–8^ including the Danish national birth registry.^9^ Because DS is one of the most common genetic birth defects with a worldwide incidence of 1 in 800–1000 live births annually,^10,11^ strategies that prevent severe RSV disease in this high-risk group could have global impact on RSV-related mortality.

Currently, the only available strategy to protect children at risk against RSV infection is passive immunization by RSV-specific monoclonal antibodies (palivizumab). Other potential future strategies include infant and pediatric vaccination and passive immunization by maternal vaccination or by extended half-life antibodies.^12^ Several vaccine candidates are under clinical development,^13^ and a recent phase III maternal vaccine trial has shown promising results.^14,15^

To guide policy makers on the implementation of perinatal immunization strategies, the efficacy of these strategies for the different target populations needs to be investigated. For this, information on age distribution and clinical manifestations of RSV-related mortality is of major importance.^16,17^ Data on global RSV-related mortality in children with DS are absent. The aim of this study was to describe demographic and clinical characteristics of children with DS who died in hospital with RSV-confirmed infection younger than 5 years of age.

## MATERIALS AND METHODS

### Study Design and Study Population

We performed a retrospective study involving a subgroup of children with DS derived from the RSV Global Online Database (GOLD). In short, RSV GOLD is an ongoing global study that retrospectively analyzes individual data of children who died with RSV infection. Collaborators can share cases through a link to an online questionnaire in Research Online, an electronic data capture platform.^18^ Each case is validated with the collaborator by RSV GOLD team members to ensure data quality. Detailed description of data collection and validation and primary results have already been published.^3^ The initial results (GOLD I) included data from January 1, 1995, to October 31, 2015. The study was extended (GOLD II) and includes data from nosocomial infections as well as community-acquired infections up to 2020. For the current study, we analyzed cases of children with DS from GOLD I (n = 17) and new cases (GOLD II, n = 36).

### Data Collection and Case Definition

Included were children with DS who died younger than 5 years of age with laboratory-confirmed RSV infection. Mortality cases that occurred before 1995 were excluded. We extracted the following demographic characteristics: gender, gestational age in weeks, prematurity and country of origin. Prematurity was defined as gestational age <37 weeks. Country of origin was categorized as low-income, lower-middle-income, upper-middle-income, and high-income based on the World Bank classifications for 2020.^19^ Clinical characteristics consisted of presenting signs and symptoms, length of stay in hospital, admission to an intensive care unit (ICU), ICU length of stay, the need for mechanical ventilation and the presence of comorbidities. The following comorbidities were distinguished: CHD, chronic lung disease, pulmonary hypertension, congenital hypothyroidism, immune disorder, neuromuscular disorder and cancer. When data for comorbidities or prematurity were not recorded, we performed data validation and inquired with the collaborators whether this information was available. If this was not the case, we assumed that the children were born term and had no comorbidities besides DS. We compared demographic and clinical characteristics of children with DS with and without comorbidities. We also compared characteristics of children with DS from GOLD I and II to children without DS from GOLD I.

### Age Distribution at Time of Death

RSV GOLD was initiated to inform the maternal vaccine program. A maternal vaccine will provide only temporary protection of approximately 3 months after birth due to the gradual decline of maternally derived antibodies.^20^ Therefore, we determined the proportion of children who died within the first 3 months after birth that could have been potentially prevented by a maternal vaccine. We distinguished additional risk factors for severe RSV disease, consisting of prematurity and the following comorbidities: CHD, chronic lung disease, immune disorder and cancer. Age distribution at time of death was compared between children with DS and additional factors for severe RSV disease and children with DS without additional risk factors. We performed a sensitivity analysis excluding cases with missing data for prematurity. Furthermore, we compared the age distribution at time of death between children with DS and without DS.

### Statistical Analysis

Continuous variables are presented as the median with interquartile ranges (IQRs). Categorical variables are presented as numbers and percentages. A χ^2^ test or Fisher exact test was used to determine statistical significance between groups in case of dichotomous parameters. A Mann-Whitney *U* test was used for all continuous data, assuming a non-normal distribution. A *P* value <0.05 was considered statistically significant. SPSS (version 21.0; IBM Corp, Armonk, NY) was used for all analyses.

### Ethics Statement

Because this is a retrospective study in which only anonymized secondary patient data were involved, parental informed consent was not deemed necessary by the institutional research board of the University Medical Centre Utrecht. Ethics approval was obtained for a few individual collaborating institutes when needed.

## RESULTS

**FIGURE 1.**
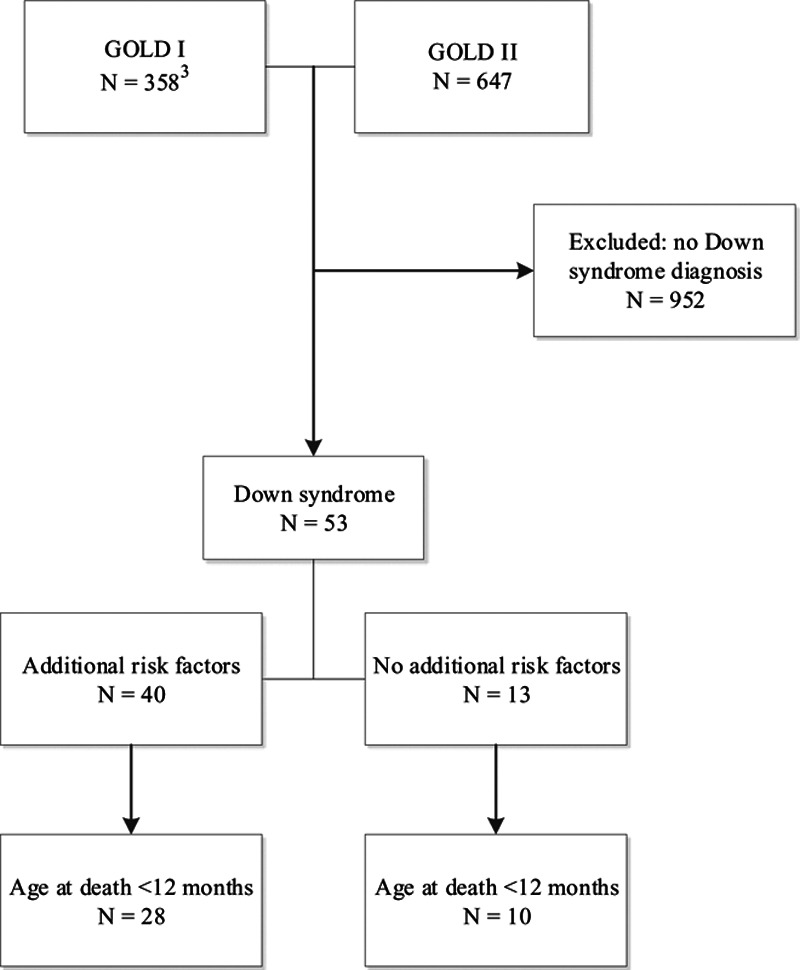
Inclusion of children with Down syndrome from the RSV GOLD I and II registry.

Fifty-three children with DS who died younger than 5 years of age with laboratory-confirmed severe RSV infection were reported to the RSV GOLD registry between January 1, 1995, and June 21, 2019 (Fig. 1). The majority of cases occurred after 2010; median year of death was 2012.

### Demographic and Clinical Characteristics

Reported children originated from 20 different countries across the world (Table, Supplemental Digital Content 1, http://links.lww.com/INF/D890; Figure, Supplemental Digital Content 2, http://links.lww.com/INF/D891. Five (9.4%) children were from low-income or lower-middle-income countries, 27 (50.9%) children were from upper-middle-income countries and 22 (41.5%) children were from high-income countries. Median reported gestational age was 37 weeks (IQR: 35.8–38.2). Thirteen (24.5%) children were born prematurely (data on prematurity were missing for 14/53 children). The majority of children with DS were male (n = 31, 58.5%). Main presenting signs and symptoms were difficulty with breathing (n = 35/46, 76.1%) and coughing (n = 27/43, 62.8%). Median length of hospital stay was 13 days (IQR: 6.8–21.0). Forty (n = 40/48, 83.3%) children were admitted to an ICU, and 33 (n = 33/51, 64.7%) children required mechanical ventilation for a median duration of 10 days (IQR: 6.0–16.0). Median age at time of RSV-related death was 6 months (IQR: 3.0–12.0). Comorbidities were reported for 39 (73.6%) children with DS. Nine children had more than 1 comorbidity (Table 1). Seven children had a confirmed nosocomial RSV infection. Data on administration of palivizumab were available for 13 children. Of these, only 1 child with DS and nonhemodynamically significant CHD had received palivizumab prophylaxis, consisting of 1 dose at the beginning of the RSV season. The child died at the end of the RSV season at the age of 5 months.

**TABLE 1.**
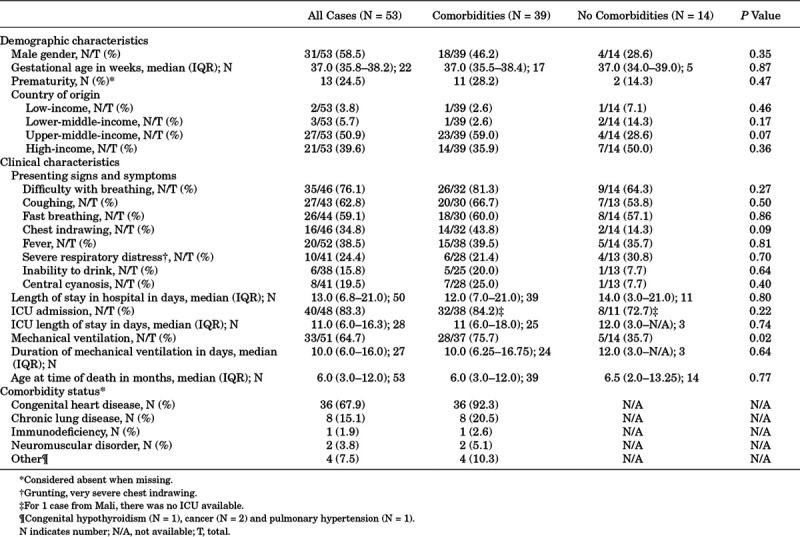
Demographic Characteristics, Clinical Characteristics and Comorbidity Status of Children With Down Syndrome Under 5 Years of Age Who Died With Laboratory-Confirmed RSV Infection

### Proportion of RSV-related Deaths 3 Months of age or Younger

**FIGURE 2.**
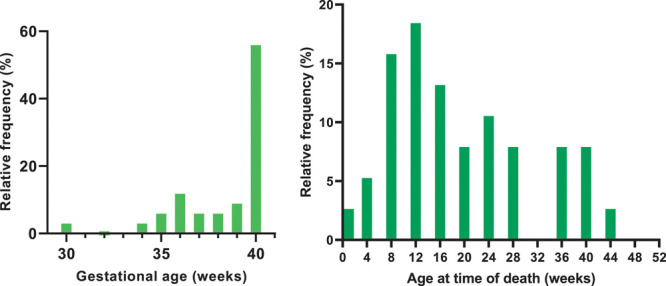
Distribution of gestational age (GA) (N = 34*) and age in weeks at time of RSV-related death for children with Down syndrome <12 months (N = 38). *Four cases were excluded because they were born prematurely with unknown GA.

Thirty-eight (71.7%) children were younger than 12 months at time of RSV-related death and 10 (18.9% of total) of these children were younger than 3 months at time of death. The distributions for gestational age and age at RSV-related death in children with DS younger than 12 months are shown in Figure 2. The distributions for gestational age and age at RSV-related death in all reported children with DS are shown in Figure, Supplemental Digital Content 3, http://links.lww.com/INF/D892.

### Comparison Between Children With DS With and Without Risk Factors for Severe RSV Disease

**FIGURE 3.**
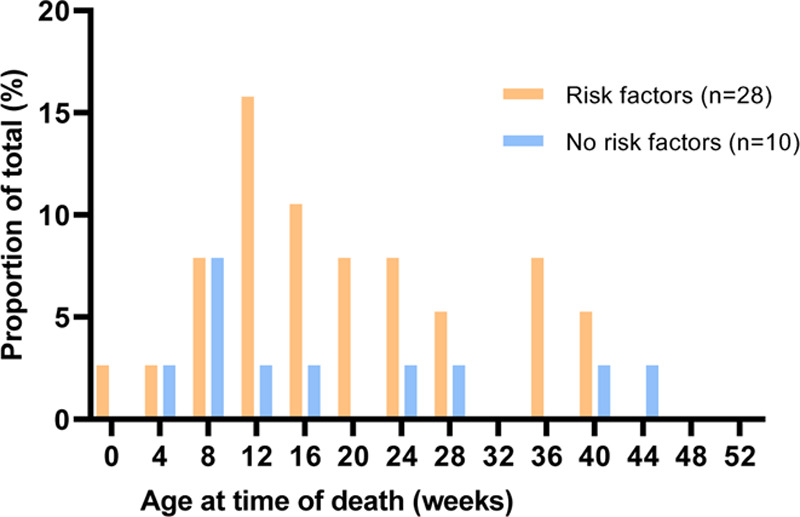
Distribution of age in weeks at time of RSV-related death for children with Down syndrome with (N = 28) and without (N = 10) additional risk factors for severe RSV disease <12 months.

In total, 13 (24.5%) children were born term and had no risk factors for severe RSV disease other than DS. We compared age distribution at time of RSV-related death between children with DS younger than 12 months with (n = 28) and without (n = 10) additional risk factors for severe RSV disease (Fig. 3) and for all reported cases (Figure, Supplemental Digital Content 4, http://links.lww.com/INF/D893. There was no significant difference in age at time of death between groups (*P* = 0.74 and *P* = 0.56, respectively). A sensitivity analysis excluding cases with missing data for gestational age and no additional risk factors (n = 5) gave a similar result (*P* = 0.59).

### Comparison Between Children With and Without DS

In Table, Supplemental Digital Content 5, http://links.lww.com/INF/D894, we compared characteristics of children with DS and without DS (GOLD I, previously published).^3^ Children with DS had significantly less presenting signs and symptoms of respiratory tract infection (*P* < 0.0005) but were hospitalized longer [median length of stay, 13 days (IQR: 6.8–21.0) vs. 8 days (IQR: 3.0–18.5), *P* = 0.005]. This difference was still significant (*P* = 0.02) when excluding children with confirmed nosocomial infection [median length of stay, 12 days (IQR: 6.0–20.0)]. There was no statistical difference in age at time of death between groups (*P* = 0.64, Figure, Supplemental Digital Content 6, http://links.lww.com/INF/D895), also when excluding children with confirmed nosocomial infection. We subsequently analyzed children with and without DS, without additional risk factors for severe RSV disease (Figure, Supplemental Digital Content 7, http://links.lww.com/INF/D896). Again, there was no statistically significant difference in age at time of death between groups (*P* = 0.95).

## DISCUSSION

This study is the first global case series of children with DS who died with RSV-confirmed infection. We evaluated the demographic and clinical characteristics of these children and found that median age at death was 6 months. Median age at death was similar in children with and without risk factors other than DS for severe RSV disease and similar in children with and without DS.

This study adds to the existing literature by describing demographic and clinical characteristics from 53 children with DS from 20 different countries. LRTIs are the primary cause of hospitalization^21^ and form a major cause of mortality in children with DS.^22^ We have previously shown that DS is an independent risk factor for severe RSV infection,^4^ and this was confirmed by others.^5–9^ In 3 meta-analyses, the relative risk for RSV hospitalization for children with DS was found to be 6- to 8-fold higher compared with children without DS,^6,22,23^ and the relative risk of mortality was approximately 9-fold higher for children with DS.^22^ Increased susceptibility for severe LRTIs in this group may be explained by 3 factors: first, DS-associated airway malformations, such as laryngotracheomalacia, alveolar and pulmonary hypoplasia^24,25^; second, DS-associated comorbidities, such as hemodynamically significant CHD, pulmonary hypertension, generalized hypotonia, swallowing dysfunction with increased risk of aspiration; and third, immunologic impairments, such as decreased NK-cell activity, abnormal thymus function, lower numbers of T and B cells and decreased T-cell proliferation and cytotoxicity.^26,27^ Altogether, these factors accumulate to an increased risk of death in case of RSV infection.

When comparing children with DS to children without DS, we found that children with DS were hospitalized longer than children without DS. A possible explanation could be that physicians may tend to admit children with DS and respiratory tract infection (RTI) quicker than other children with RTI due to the presence of the above-mentioned risk factors for severe respiratory disease.

Prevention of RSV-related morbidity and mortality is needed for children with DS. To date, the only available RSV prophylaxis is palivizumab, a humanized monoclonal antibody which is administered monthly before the start of the RSV season.^28^ The efficacy of palivizumab is firmly established^29,30^ and routinely recommended for children with CHD, chronic lung disease or born prematurely. Some pediatricians advocate offering palivizumab to every child with DS up to 2 years of age,^31,32^ while others await the development of an RSV vaccine. Currently, most prophylaxis guidelines do not yet recommend palivizumab for children with DS^33,34^ because there are insufficient studies that address the efficacy and cost-effectiveness of palivizumab in children with DS without additional risk factors for severe RSV disease.^35^ The American Academy of Pediatrics states that children with DS without additional risk factors are generally older at RSV-related hospitalization compared with children with additional risk factors (median age, 9 vs. 4 months).^36^ Therefore, immunoprophylaxis for the first year of life would be of limited effect.^33^ On the other hand, some countries have included DS as possible indication for palivizumab prophylaxis in their guidelines, acknowledging that randomized controlled trials are challenging to conduct due to the projected large sample size and ethical concerns.^37^ Nevertheless, palivizumab is costly and therefore barely available in low-income and lower-middle-income countries.

The results of the first maternal RSV vaccine candidate reaching phase 3 showed prevention of severe RSV infection in babies born to vaccinated mothers, but the trial did not meet its primary endpoint.^14^ In the present study, approximately one-fifth of cases were younger than 3 months at time of death. This implies that maternal vaccination may not sufficiently protect all children with DS against life-threatening RSV infection, given the fact that the level of maternally acquired RSV-specific antibodies declines over time after birth and will only provide temporary protection.^17,38,39^ As an alternative to palivizumab, which requires multiple dosing during the RSV season and is therefore costly, an extended half-life monoclonal antibody has been developed (nirsevimab, previously MEDI8897). This highly potent antibody has shown promising results in a phase IIb trial and was recently granted Breakthrough Therapy Designation by the United States Food and Drug Administration.^40^

The strengths of this study consist of the global representation of RSV-related mortality in children with DS in our mortality registry. Large studies such as RSV GOLD are essential to obtain sufficient global data for this high-risk group. We have obtained good data quality by verifying each case directly with the RSV GOLD collaborators. Furthermore, we differentiated between children with DS with and without comorbidities and risk factors for severe RSV disease.

There are also limitations to this study. First, only 53 children with DS were reported to the RSV GOLD registry. This is a small proportion of all children with DS who died with RSV worldwide. We could not estimate the actual burden of RSV-related death in this high-risk group because the incidence of DS was not available for most countries. In addition, not all RSV-related mortality cases that occurred in these countries have been shared with the GOLD registry. However, we believe that children with DS are overrepresented in the RSV GOLD mortality registry, because the proportion of children with DS reported to the registry is larger than the prevalence of DS in the general population (1:19 reported GOLD cases vs. 1:1200 in the population^41^). Second, because we collected data from 20 different countries over a period of more than 25 years, quality of care might have differed substantially between cases. Third, data for prematurity and (severity of) comorbidities were often incomplete, such as type of CHD and whether the child underwent cardiac surgery before RSV infection. This could have resulted in an underestimation of the proportion of children with DS with additional comorbidities and children who were born prematurely. However, the estimated general incidence of CHD in children with DS is comparable to our results (50%–66.6%^42,43^ vs. 67.9%, respectively). Furthermore, since DS was already reported for these children, it is more likely that other comorbidities, if present, would also have been reported. Moreover, a sensitivity analysis excluding cases with missing data for gestational age gave similar results. Fourth, because nosocomial RSV infection and stem cell transplantation were exclusion criteria for GOLD I, the proportion of children with nosocomial RSV infections and of those who received stem cell transplantation is underrepresented in this study. Lastly, this study hardly represents children with DS from low-income and lower-middle-income countries. This is most likely due to a lack of RSV testing and to limited access to healthcare—leading to both early death in children with DS with underlying comorbidities before they become RSV infected and to RSV-related death in the community.

In conclusion, children with DS are at increased risk of RSV-related death and need adequate protection against RSV infection. Considering median age at death, maternal vaccination will not be sufficient for this high-risk group.

## ACKNOWLEDGMENTS

We thank our collaborators of the Respiratory Syncytial Virus (RSV) Global Online Database (GOLD) study group for taking the time and effort to share cases with the RSV GOLD registry. We also thank Heather Zar, Daniel Feikin, Johan Vekemans, Naveen Thacker, Nicole Derksen, Michael Boele van Hensbroek and Christopher Gill for their scientific input as (former) members of the RSV GOLD-independent scientific advisory board. Furthermore, we thank Prachi Vora, Niteen Wairagkar, Padmini Srikantiah and Leyla Kragten-Tabatabaie for their scientific advice. Lastly, we thank our (former) members of the RSV GOLD operations team Ichelle van Roessel, Rogina Roebaar, Dunja Scheepmaker, Trisja Boom, Femke Vernooij, Beverly de Leeuw, Jasper van der Kemp, Renske Bijl, Sophia van der Graaf, Sangeeta Bisheshar, Dora van Duijvendijk, Sophie Croon, Insaf Duale, Junhua Fang and Issam el Mansori for their hard work, enthusiasm and valuable contributions.

## Supplementary Material



## RSV GOLD COLLABORATORS

The RSV GOLD collaborators for this study are as follows: Asuncion Mejias, MD, PhD, and Octavio Ramilo, MD: Division of Infectious Diseases, Department of Pediatrics, Center for Vaccines and Immunity at Nationwide Children’s Hospital, Columbus, Ohio; Kentigern Thorburn, MD, and Paul S. McNamara, MD, PhD: Alder Hey Children’s Hospital/The University of Liverpool, Liverpool, United Kingdom; Hsin Chi, PhD: Department of Pediatric Infectious Disease, MacKay Children’s Hospital, Taipei, Taiwan; Uzma Bashir Aamir: Department of Virology, National Institute of Health, Islamabad, Pakistan; Márcia R. Pires, and Fernanda de-Paris: Molecular Biology Laboratory and Infection Control Commission, Hospital de Clínicas de Porto Alegre, Bairro Santa Cecília, Porto Alegre, Brazil; Angela Gentile, MD, and María F. Lucion, MD: Ricardo Gutiérrez Children’s Hospital, Buenos Aires, Argentina; Sergio de Andrade, Nishioka MD, PhD: Ministry of Health, Brasilia, Brazil; on behalf of the Ministry of Health (Brazil) Orli Megged, MD: Shaare Zedek Medical Center, Jerusalem, Israel; Danielle Hessong, MPH, and Eric A. F. Simões, MD, PhD: Children’s Hospital Colorado, Colorado; Shaun K. Morris, MD, MPH, and Waison Wong, MD: Division of Infectious Diseases, The Hospital for Sick Children, Toronto, Ontario, Canada; Thyyar M. Ravindranath, MD: Columbia University Vagelos College of Physicians and Surgeons and NewYork- Presbyterian Morgan Stanley Children’s Hospital New York, New York, New York; Alfredo C. Bruno, MSc: Instituto Nacional de Investigación en Salud Publica, Guayaquil, Ecuador and Universidad Agraria del Ecuador, Guayaquil, Ecuador; Domenica de Mora, MSc: Instituto Nacional de Investigación en Salud Publica, Guayaquil, Ecuador; Jenny Ojeda, MSc, and Lida Zamora, MSc: Ministerio de Salud Pública del Ecuador, Quito, Ecuador; Simon Nadel, PhD, and Marwa Ghazaly, MD, PhD: St Mary’s Hospital, Imperial NHS, London, United Kingdom; Satoshi Kusuda, MD, PhD: Kyorin University, Tokyo, Japan; Francisco J. Elola, MD, PhD, and Manuel Sánchez Luna, MD, PhD: Fundación Instituto para la Mejora de la Asistencia Sanitaria/Research Institute Gregorio Maranon, Madrid, Spain; Srđan Roglić, MD, PhD: University Hospital for Infectious Diseases, Zagreb, Croatia; Irini Eleftheriou, MD, and Maria Tsolia, MD, PhD: Children’s Hospital “P. & A. kyirakou”, Athens, Greece; Nasamon Wanlapakorn, MD, PhD: Faculty of Medicine, Chulalongkorn University, Bangkok, Thailand; Ghassan S. Dbaibo, MD, and Rima Hanna Wakim, MD: American University of Beirut Medical Center, Beirut, Lebanon; D. James Nokes, PhD, and Grieven P. Otieno, BSc: Kenya Medical Research Institute, Wellcome Trust Research Programme, Centre for Geographic Medicine Research - Coast, Kilifi, Kenya; Dario Prais, MD: Schneider Children`s Medical Center of Israel, Petah Tikva, Israel and Sackler Faculty of Medicine, Tel Aviv University, Tel Aviv, Israel; Heloisa Ihle Garcia Giamberardino, MD, and Jane M. Webler: Hospital Pequeño Principe, Curitiba, Brazil; Mauricio T. Caballero, MD, and Fernando P. Polack, MD: Fundación Infant, Buenos Aires, Argentina; Daniel E. Noyola, MD, PhD: Universidad Autonoma de San Luis Potosí, San Luis Potosí, Mexico; Rashmi Das, MD: All India Institute of Medical Sciences (AIIMS), Bhubaneswar, India; and Katherine L. O’Brien, MD: International Vaccine Access Center, Johns Hopkins Bloomberg School of Public Health, Baltimore, Maryland; on behalf of the PERCH Study Group.
