# Supplementary material for: Respiratory Syncytial Virus-related Death in Children With Down Syndrome: The RSV GOLD Study
Source: Pediatr Infect Dis J. 2020 Apr 24;39(8):665–70. doi: 10.1097/INF.0000000000002666 (PMC7360096; doi:10.1097/INF.0000000000002666)
Supplement: Supplementary file 1 [file inf-39-0665-s001.docx]

| **Supplemental Digital Content 1**. Country where RSV-related death occurred | |
| --- | --- |
| **Country**  (N = 20) | **Children with Down syndrome and RSV-related death**  (N = 53) |
| **Low-income** | **2 (3.8%)** |
| Mali | 2 (3.8%) |
| **Middle-income** | **30 (56.6%)** |
| ***Lower-middle-income*** | ***3 (5.7%)*** |
| India | 1 (1.9%) |
| Kenya | 1 (1.9%) |
| Pakistan | 1 (1.9%) |
| ***Upper-middle-income*** | ***27 (50.9%)*** |
| Brazil | 11 (20.8%) |
| Argentina | 9 (17.0%) |
| Ecuador | 3 (5.7%) |
| Mexico | 1 (1.9%) |
| Lebanon | 1 (1.9%) |
| South Africa | 1 (1.9%) |
| Thailand | 1 (1.9%) |
| **High-income** | **22 (41.5%)** |
| Spain | 4 (7.5%) |
| Canada | 4 (7.5%) |
| United States | 4 (7.5%) |
| United Kingdom | 2 (3.8%) |
| Taiwan | 2 (3.8%) |
| Israel | 2 (3.8%) |
| Japan | 1 (1.9%) |
| Croatia | 1 (1.9%) |
| Greece | 1 (1.9%) |
