# Supplementary material for: Respiratory Syncytial Virus-related Death in Children With Down Syndrome: The RSV GOLD Study
Source: Pediatr Infect Dis J. 2020 Apr 24;39(8):665–70. doi: 10.1097/INF.0000000000002666 (PMC7360096; doi:10.1097/INF.0000000000002666)
Supplement: Supplementary file 2 [file inf-39-0665-s002.docx]

**Supplemental Digital Content 2.** Global distribution of included children with Down syndrome and RSV-related death


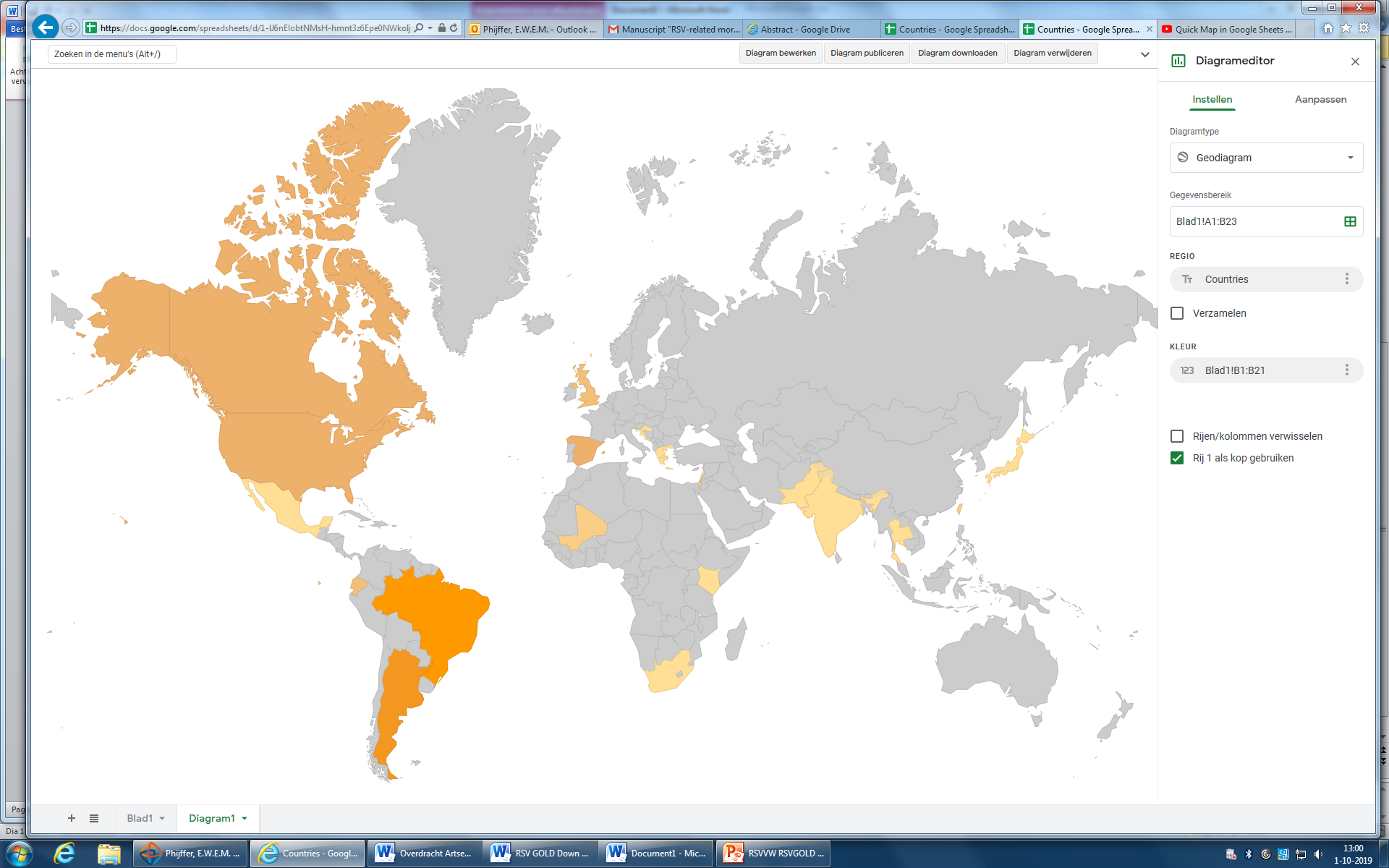


**1 ..**

**1 ..**

**2 ..**

**1 ..**

**1 ..**

**2 ..**

**1 ..**

**1 ..**

**4 ..**

**1 ..**

**2 ..**

**2 ..**

**11 ..**

**9 ..**

**3 ..**

**1 ..**

**1 ..**

**1 ..**

**4 ..**

**4 ..**
