# Supplementary material for: Respiratory Syncytial Virus-related Death in Children With Down Syndrome: The RSV GOLD Study
Source: Pediatr Infect Dis J. 2020 Apr 24;39(8):665–70. doi: 10.1097/INF.0000000000002666 (PMC7360096; doi:10.1097/INF.0000000000002666)
Supplement: Supplementary file 4 [file inf-39-0665-s004.docx]

**Supplemental Figure 3**. Distribution of age in months at time of RSV-related death for children with Down syndrome with (N = 40) and without (N = 13) additional risk factors for severe RSV disease
