# Supplementary material for: Respiratory Syncytial Virus-related Death in Children With Down Syndrome: The RSV GOLD Study
Source: Pediatr Infect Dis J. 2020 Apr 24;39(8):665–70. doi: 10.1097/INF.0000000000002666 (PMC7360096; doi:10.1097/INF.0000000000002666)
Supplement: Supplementary file 5 [file inf-39-0665-s005.docx]

| **Supplemental Digital Content 5**. Demographic characteristics, clinical characteristics and comorbidity status of children with (N = 53) and without (N = 342) Down syndrome under 5 years of age who died with laboratory-confirmed RSV infection | | | | |
| --- | --- | --- | --- | --- |
|  | | Children with DS  (N = 53) | Children without DS  (N = 342) | P-value |
| **Demographic characteristics** | |  |  |  |
| Female gender, N/T (%) | | 22/53 (41.5) | 160/342 (46.8) | 0.47 |
| Gestational age in weeks, median (IQR); N | | 37.0 (35.8-38.2); 22 | 38.0 (33.5-40.0); 133 | 0.21 |
| Prematurity, N (%)^a^ | | 13 (24.5) | 61 (23.1); 264 | 0.25 |
| Country  of origin | Low-income, N/T (%) | 2/53 (3.8) | 5/342 (1.5) | 0.24 |
|  | Lower-middle-income, N/T (%) | 3/53 (5.7) | 109/342 (31.9) | <0.0005 |
|  | Upper middle-income, N/T (%) | 27/53 (50.9) | 145/342 (42.4) | 0.24 |
|  | High-income, N/T (%) | 21/53 (39.6) | 83/342 (24.3) | 0.02 |
| **Clinical characteristics** | |  |  |  |
| Presenting signs and symptoms | Difficulty with breathing, N/T (%) | 35/46 (76.1) | 212/221 (95.9) | <0.0005 |
|  | Coughing, N/T (%) | 27/43 (62.8) | 209/211 (99.1) | <0.0005 |
|  | Fast breathing, N/T (%) | 26/44 (59.1) | 152/170 (89.4) | <0.0005 |
|  | Chest indrawing, N/T (%) | 16/46 (34.8) | 138/159 (86.8) | <0.0005 |
|  | Fever, N/T (%) | 20/52 (38.5) | 5/312 (1.6) | <0.0005 |
|  | Severe respiratory distress, N/T (%) | 10/41 (24.4) | 72/94 (76.6) | <0.0005 |
|  | Inability to drink, N/T (%) | 6/38 (15.8) | 70/113 (61.9) | <0.0005 |
|  | Central cyanosis, N/T (%) | 8/41 (19.5) | 33/72 (45.8) | 0.005 |
| Length of stay in hospital in days, median (IQR); N | | 13.0 (6.8-21.0); 50 | 8.0 (3.0-18.5); 333 | 0.005 |
| ICU admission, N/T (%) | | 40/48 (83.3) | 191/232 (82.3) | 0.87 |
| ICU length of stay in days, median (IQR); N | | 11.0 (6.0-16.3); 28 | 10.5 (4.0-25.8); 176 | 0.70 |
| Mechanical ventilation, N/T (%) | | 33/51 (64.7) | 184/315 (58.4) | 0.40 |
| Duration of mechanical ventilation in days, median (IQR); N | | 10.0 (6.0-16.0); 27 | 10.0 (4.0-24.0); 157 | 0.90 |
| Age at time of death in months, median (IQR); N | | 6.0 (3.0-12.0); 53 | 5.6 (2.4-12.3); 342 | 0.64 |
| **Comorbidity status^a^** | |  |  |  |
| Congenital heart disease, N (%) | | 36 (67.9) | 61 (17.8) | <0.0005 |
| Chronic lung disease, N (%) | | 8 (15.1) | 44 (12.9) | 0.24 |
| Immunodeficiency, N (%) | | 1 (1.9) | 8 (2.3) | 0.69 |
| Neuromuscular disorder, N (%) | | 2 (3.8) | 23 (6.7) | 0.08 |
| ^a^Considered absent when missing  ^b^e.g. grunting, very severe chest indrawing  N, number; T, total | | | | |
