# Supplementary material for: Respiratory Syncytial Virus-related Death in Children With Down Syndrome: The RSV GOLD Study
Source: Pediatr Infect Dis J. 2020 Apr 24;39(8):665–70. doi: 10.1097/INF.0000000000002666 (PMC7360096; doi:10.1097/INF.0000000000002666)
Supplement: Supplementary file 6 [file inf-39-0665-s006.docx]

**Supplemental Figure 4**. Distribution of age in months at time of RSV-related death for children with Down syndrome (N = 53) and children without Down syndrome (N = 342)

*There was no statistical significant difference in median age at death (p = 0.641).
