# Supplementary material for: Respiratory Syncytial Virus-related Death in Children With Down Syndrome: The RSV GOLD Study
Source: Pediatr Infect Dis J. 2020 Apr 24;39(8):665–70. doi: 10.1097/INF.0000000000002666 (PMC7360096; doi:10.1097/INF.0000000000002666)
Supplement: Supplementary file 7 [file inf-39-0665-s007.docx]

**Supplemental Figure 5**. Distribution of age in months at time of RSV-related death for children without additional risk factors for severe RSV disease, Down syndrome (N = 13) versus no Down syndrome (N = 203)

* There was no statistical significant difference in median age at death (p = 0.945).
